# High-Altitude Balloon-Based Sensor System Design and Implementation

**DOI:** 10.3390/s20072080

**Published:** 2020-04-07

**Authors:** Zhanchao Wang, Min Huang, Lulu Qian, Baowei Zhao, Guangming Wang

**Affiliations:** 1 Aerospace Information Research Institude, Chinese Academy of Sciences, Beijing 100094, China; huangmin@aoe.ac.cn; 2 School of Optoelectronics, University of Chinese Academy of Sciences, Beijing 100049, China; qianlulu@aoe.ac.cn (L.Q.); zhaobaowei@aoe.ac.cn (B.Z.); wanggm@aircas.ac.cn (G.W.); 3 Key Laboratory of computational Optical Imaging Technology, Chinese Academy of Sciences, Beijing 100094, China

**Keywords:** high-altitude balloon, sensor system, Honghu Project, near space

## Abstract

As a kind of large-scale unmanned aerial vehicle, a high-altitude balloon can carry a large load up to tens of kilometers in the near space for a long time, which brings a new way for the stratosphere atmospheric detection. In order to provide a suitable working environment for the near-space detection load, it is necessary to design a sensor system based on a high-altitude balloon, which is used to provide environmental temperature, height position, and attitude information, current working, and video surveillance. The high-altitude balloon-based sensor system designed in this paper had participated in the near-space flight experiment, whose total flight time was 30 h and 53 min, and the horizontal flight time was 28 h and 58 min crossing the day and night. The high-altitude balloon-based sensor system had withstood the severe environment of the near-space during the day and night, providing accurate temperature measurement, real-time altitude position and attitude data acquisition, reliable current monitoring, and comprehensive video surveillance. In the next three years, the high-altitude balloon-based sensor system developed in this paper will continue to participate in the experiment and provide support for more detection loads.

## 1. Introduction

Near space refers to the earth space with an altitude between 20 Km to 100 Km, and it is a very complex field, which involves many subjects, such as atmospheric science, environmental science, biological science, and physics [1,2]. However, the development and utilization of the near-space are far less than that of the traditional airspace and satellite orbit space. The traditional aircraft can hardly fly up to the near-space, and the satellites in space are not easy to detect down the near-space [3]. Therefore, near space is a new research hotspot that worth more observation and detection. A high-altitude balloon is one of the near-space exploration vehicles, and it has many advantages [4]. First of all, a high-altitude balloon can achieve low-cost sustained flight for months or even years. Compared with other air vehicles, the high-altitude balloon has long endurance time, which can achieve sustained and wider coverage for regional observation and detection [5,6,7]. Secondly, compared with satellite, a high-altitude balloon can take more and heavier loads, and increase the observation accuracy and range, for example, the spatial resolution and the signal strength of the optical observation load in near space can be increased greatly [8]. Thirdly, the payloads in the near space with high altitude balloons can be recycled with low cost and low risk, which is difficult to achieve by satellites.

Since the high-altitude balloon is floated in the near space athe nd the detection payloads are in a harsh environment [9]. In order to provide a suitable working environment for the near-space detection load, it is necessary to design a sensor system based on the high-altitude balloon, which is used to provide environmental temperature, height position, and attitude information, working current and video surveillance [10]. The high-altitude balloon-based sensor system designed in this paper had participated in the near-space flight experiment, whose total flight time was 30 h and 53 min, and the horizontal flight time was 28 h and 58 min crossing the day and night. The high-altitude balloon-based sensor system had withstood the severe environmental during day and night, providing accurate temperature measurement, real-time altitude position and attitude data acquisition, reliable current monitoring, and comprehensive video surveillance.

The second part of this paper mainly introduces the high-altitude balloon-based sensor system design, including an inertial navigation sensor system, temperature sensor system, image surveillance sensor system and work current sensor system. The third part focuses on the experimental results; finally, the conclusion and discussion are provided in the fourth part.

## 2. High-altitude Balloon Sensor System Methods

### 2.1. System Overview

**Figure 1:**
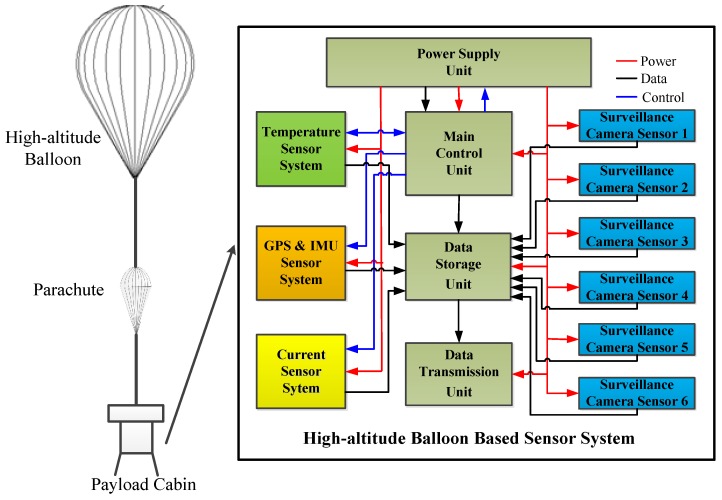
Interior block diagram of the biological experiment cabin.

In order to provide a suitable working environment for the near-space detection load, it is necessary to design a sensor system based on a high-altitude balloon, which is used to provide environmental temperature, height position, and attitude information, working current and video surveillance. The high-altitude balloon-based sensor system design, including inertial navigation sensor system, temperature sensor system, image surveillance sensor system, and work current sensor system, is shown in Figure 1.

The high-altitude balloon used in the experiment was a zero-pressure balloon with a volume of 20,000 m^3^, and the overall payload capacity was 325 Kg. There is a parachute between the high-altitude balloon and payload cabin, when the flat flying experiment is finished, the line between the parachute and the balloon is cut off. After that, the balloon rises up and bursts, the payload cabin falls, and the parachute opens. In addition to the high-altitude balloon-based sensor system, there are other equipment in the payload cabin, such as the power supply unit, main control unit, data storage unit, and data transmission unit.

### 2.2. Inertial Navigation Sensor Design

**Figure 2:**
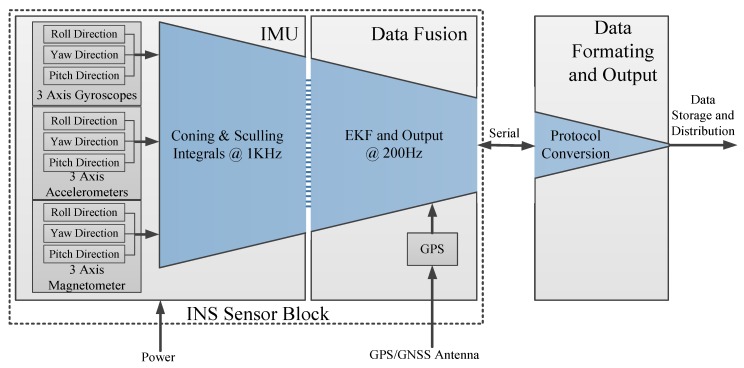
Interior block diagram of the biological experiment cabin.

In the flight process of the high-altitude balloon, it is very important to obtain its altitude, longitude, and latitude information in real time. The data from Inertial Measurement Unit (IMU)can provide a 3-axis real-time attitude of the payload cabin, and they can be used to adjust the payload cabin attitude and avoid leaning. These data are also valuable to analyze the raising processing and landing processing of the balloon. At the same time, the balloon speed, attitude, and other information are also worthy of analysis, so the attitude navigation system is in demand [11]. The inertial navigation sensor (INS) designed in this paper can provide both orientation and navigation data, which includes an IMU and runs an onboard enhanced Extended Kalman Filter (EKF). The use of Global Navigation Satellite System(GNSS) data or other aiding equipment such as odometer or internal barometric sensor can be used to provide accurate navigation data, but also to improve orientation accuracy [12]. The design block of the INS system, is shown in Figure 2; it includes IMU, data fusion unit and data formatting and output unit. The IMU embeds three high-performance, industrial-grade Micro Electro-Mechanical Systems (MEMS) accelerometers; three high-end industrial grade MEMS gyroscopes; three anisotropic magneto-resistive magnetometers.

Since IMU is the main component of the INS, the IMU used in this paper has been carefully designed to take full advantage and performance of MEMS technology. The IMU embeds 3 high-performance, industrial grade MEMS accelerometers and it is coupled with a cutting edge calibration, advanced filtering techniques and sculling integrals, these accelerometers will provide excellent performance, even in highly vibrating environment. The set of 3 high end industrial grade MEMS gyroscopes is sampled at 10 KHz. An efficient FIR filter and coning integrals computations ensures best performance in vibrating environments [13]. A set of three anisotropic magneto-resistive magnetometers is embedded within the IMU, which can provide a very high sensitivity compared to coil based technologies. The INS also embeds an industrial Global Navigation Satellite System (GNSS) receiver which can receive GPS L1 C/A, GLONASS L10F, QZSS L1 C/A and BeiDou B1 signal. This receiver has an excellent sensitivity for continuous tracking under challenging environments with a refresh rate of 5 Hz. The INS also embeds a MEMS pressure sensor, used as altimeter. This pressure sensor is fully calibrated and temperature compensated making it ideal to measure accurately absolute pressure. The item specifications of the parts in the INS are listed in Table 1.

Inertial sensors (accelerometers and gyroscopes) can provide very accurate short term motion measurements, but they suffer from drift when integration time becomes long. Some other systems such as GNSS receivers or odometer provide low-frequency measurements that can be fooled by jamming or short-term measurement errors, but these sensors provide good performance over the long term. The basic idea of Extended Kalman Filter (EKF) is to take the best of each sensor, and it includes a high-frequency prediction step using inertial sensors to precisely measure motion and navigation data [14,15,16]. The loose coupling between GPS/GNSS and the EKF allows GPS data to improve inertial sensor performance, and on the other hand, inertial data improve overall navigation performance. More than just a direct EKF implementation, the implemented algorithms include advanced error models and wrong measurement detection to ensure that the best navigation performance is provided at any time.

**Figure 3:**
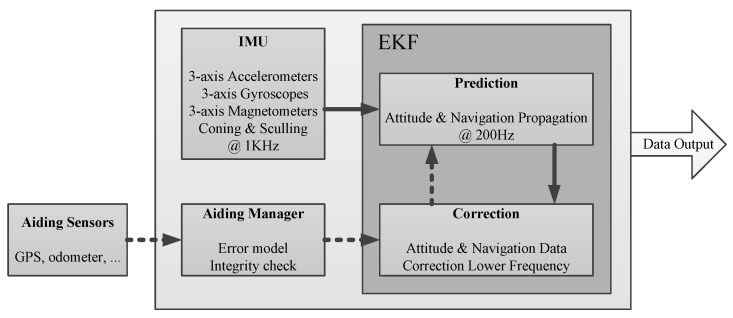
Simplified diagram block of Extended Kalman Filter (EKF).

When the aiding data from the GPS device or the odometer device becomes available, the EKF will use it to correct the current state and prevent drift. As aiding measurements are made at a lower frequency than the prediction step, a small jump can be observed after a correction is applied. This jump should be really small in normal operating conditions. A covariance matrix maintains up to date each estimated parameter error. When there is no measurement available, estimation error tends to increase; when a new measurement is received, this error will decrease. Figure 3 shows a simplified diagram block of the EKF, the IMU data, and external sensors data that are used inside the EKF to provide the navigation and orientation data.

**Figure 4:**
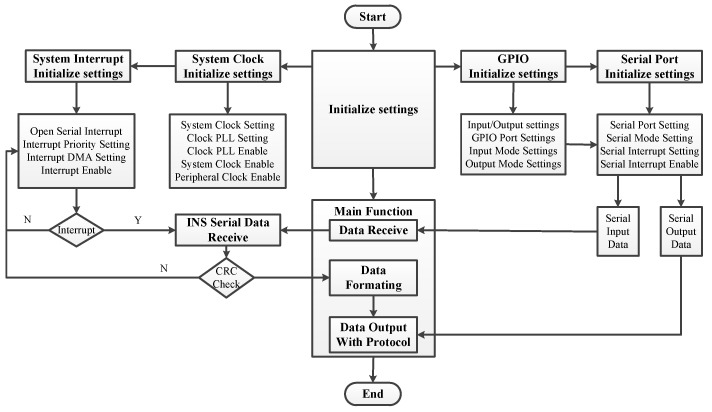
Data formatting and output processing flow chart.

Data formatting and output processing unit receives the original INS data and formats the INS data with a certain protocol and outputs the final INS data to other devices. The data formatting and output functions are finalized on the Micro Control Unit (MCU), the working flow chart of the MCU is shown in Figure 4. The MCU receives the original data from INS through serial port, and judges whether the received data is correct through CRC verification; at last, it converts the unit of the original INS data outputs the final data according to the protocol.

### 2.3. Temperature Sensor Design

**Figure 5:**
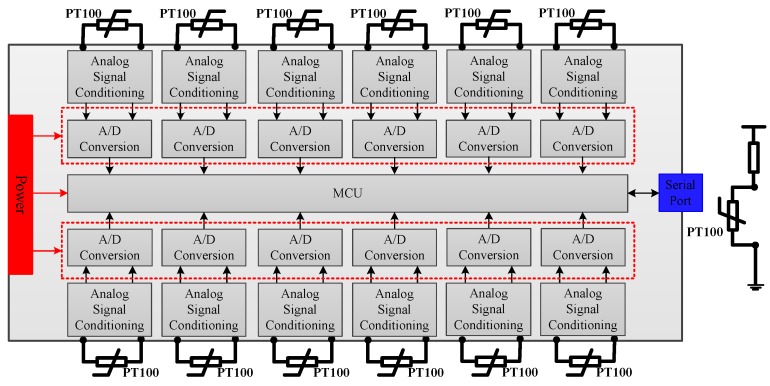
Diagram block of the temperature sensor system.

The temperature in the near-space can be very low from −50 °C to −70 °C, and it is important to measure the environment temperature around the payloads and other equipment [17]. The temperature sensor system designed in this paper adopts PT100 as a temperature sensor whose resistance changed with temperature. Resistance change on the PT100 results in the voltage change on the PT100, and the voltage can be sampled by Analog to Digital (A/D) chip. By analyzing the voltage change, the resistance of the PT100 can be calculated, thus the temperature is obtained since the resistance, and the temperature have a corresponding relationship. There are 12 PT100 to measure the temperature; the design block of the system is shown in Figure 5.

PT100 is a temperature sensed resistor, and the temperature could be calculated by measuring the resistance. The voltage on the PT100 is conditioned firstly to smooth the analog signal and adjust the voltage with the A/D chip. A/D chip used here is a 24-bit high resolution, and low power chip, whose analog input is the differential interface, and the digital output interface is the Serial Peripheral Interface (SPI). MCU is the main processing unit which is responsible for receiving 12 A/D conversion data and calculating the temperature according to the corresponding relationship between PT100 temperature and resistance value, and finally sending the temperature data through the serial port according to the protocol.

**Figure 6:**
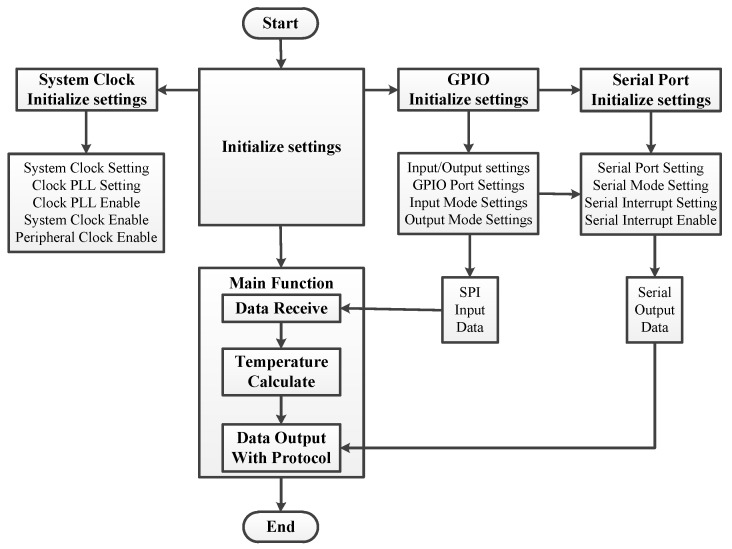
Temperature sensor processing flow chart.

Temperature sensor processing unit receives the A/D conversion data with certain protocol and calculates the temperature. The data receiving, temperature calculating, and output formatting function are finalized on the MCU; the working flow chart of the MCU is shown in Figure 6. The MCU receives the original data from 12 A/D chips through SPI and calculates the temperature data according to voltage and resistance; at last, it outputs all the final temperature data according to the protocol.

### 2.4. Image Surveillance Sensor Design

**Figure 7:**
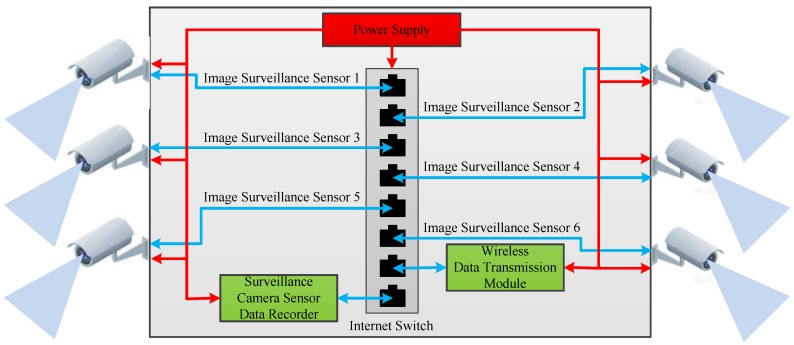
Diagram block of the image surveillance sensor system.

Image surveillance during the high-altitude balloon flight period is important, and it can provide many observation views towards payloads, balloon, and it can recorder the raising process and landing process for later analysis. Diagram block of the image surveillance sensor system is shown in Figure 6 and Figure 7 surveillance cameras are connected to the data recorder through internet switch on the one hand; and on the other hand, they are connected to the high-speed wireless data transmission module which can transmit the video data to the ground console station for surveillance.

The image surveillance sensor system contains six cameras, and they are installed inside and outside the payload cabin to record different objects. Surveillance camera 1 is installed in the payload cabin to record the downside view. Surveillance camera 2 and 3 are also installed in the payload cabin to record front view and back view, respectively. Surveillance camera 4 is installed outside the payload, and it looks upward to record the balloon state and parachute state. Surveillance camera 5 is installed near one of the payloads to record the payloads movements and state. Surveillance camera 6 is installed on the payload cabin leg to record the landing process of the payload cabin. 

### 2.5. Work Current Sensor Design

**Figure 8:**
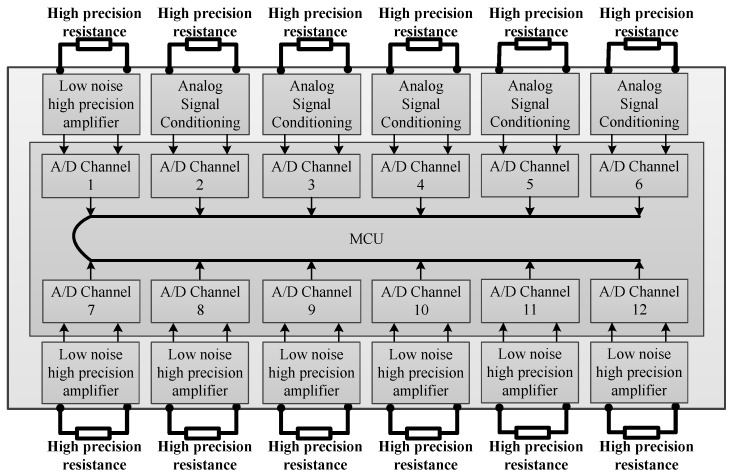
Diagram block of the work sensor system.

The work current sensor is used to monitor the working current of each payload during the high-altitude balloon flight. High-precision resistance is used to measure the current of each power supply channel since the current will produce a voltage at both ends of the resistance, as shown in Figure 8. High-precision resistance used here is 5 mΩ, 1 A current can make 5 mV voltage, which is too small to be sampled. Low noise and high precision amplifiers are used to amplify the small voltage to the suitable voltage for A/D conversion; the magnification can be from 100–1000. 

## 3. Results

**Figure 9:**
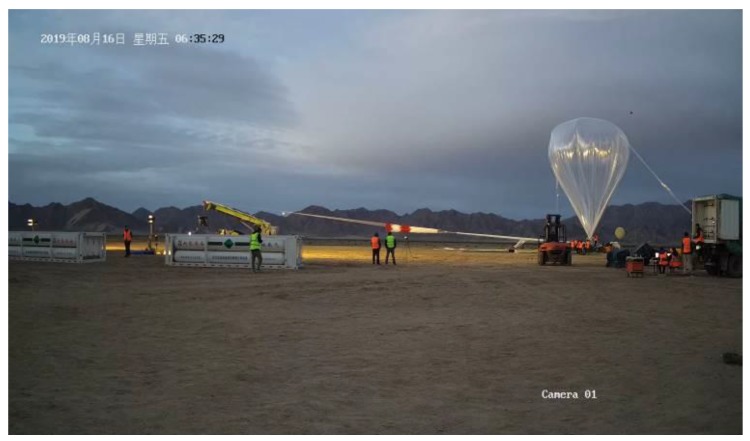
Releasing process of the high-altitude balloon.

The high-altitude balloon-based sensor system experiment was conducted on August 16, 2019, in Dachaidan district, Qinghai Tibet Plateau. The high-altitude balloon used in the experiment was a zero-pressure balloon with a volume of 20,000 m^3^, the overall capacity was 325 Kg, and the payload capacity was 205 Kg. The experiment started at 5:45 in the morning, and the balloon was released at 6:45, the releasing process is shown in Figure 9, which is taken by a ground camera. 

After 70 min for lifting, the balloon reached the zero-wind area, at an altitude about 21 Km, and the flat flying time was about 29 h as scheduled since the battery capacity almost run out, and there was no solar power supply. When the flight was over, the line between the balloon and payload cabin was cut, and the payload cabin started falling, at the same time, the parachute opened to slow down the falling speed. At last, the payload cabin landed safely, and successfully, all the payloads and sensor systems were recovered. Inertial navigation sensor recorded the altitude, longitude, latitude, and speed in three directions and attitude in three directions during the whole flight. Temperature sensors, image surveillance sensors, and current sensors worked normally and obtained precious and reliable data.

### 3.1. Inertial Navigation Sensor Result

**Figure 10:**
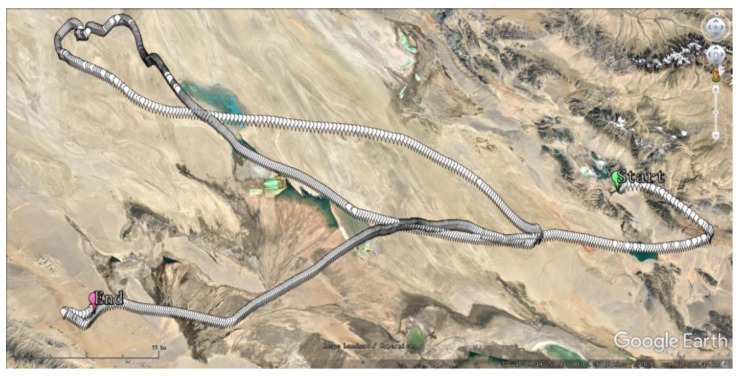
Location of the high-altitude balloon on the Google map.

The overall experiment time was more than 30 h, and the INS data was more than 108,000, considering the refresh frequency was 1 Hz. We sampled the latitude and longitude data and added on the Google map to indicate the flight path during the whole flight, as shown in Figure 10, the green point is the start point, and the pink point is the landing point, and the distance scale is 17.5 Km.

**Figure 11:**
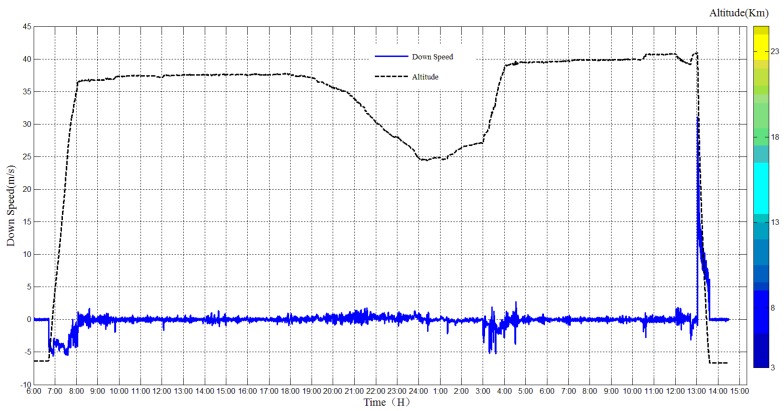
Altitude change and raising speed of the high-altitude balloon.

The altitude data and speed data are important for the analysis of the flight results, the altitude change and upward speed are plotted together, as shown in Figure 11. The raising period lasted for about one hour, and the raising speed was about 5 m/s. When the night came, the altitude dropped a lot since the low temperature caused a decrease in the buoyancy. At last, the falling period lasted for about 40 min, with the speed falling from 31 m/s–5 m/s. 

**Figure 12:**
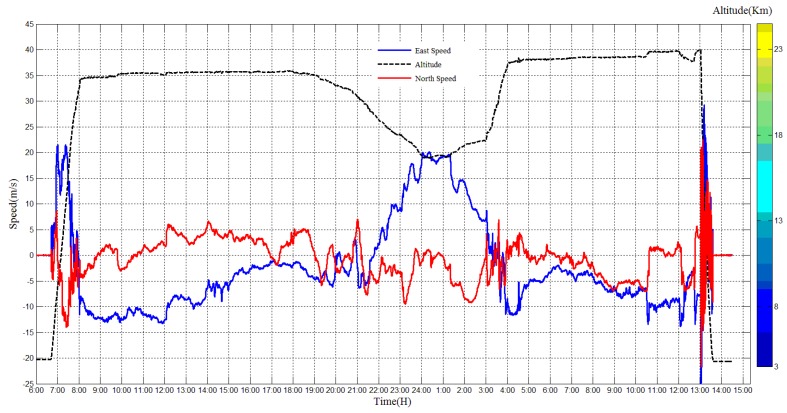
East speed and north speed of the payload cabin during the experiment.

The east direction speed and north direction speed is shown in Figure 12. It can be seen that the east speed and north speed are corresponding to the flight route in Figure 10. The blue line indicates the east direction speed, and the red line indicates the north speed. At the raising period, the balloon flew to the north-east direction, and then it turned to south-east direction. During the flat flying period, the north speed changed smaller than the east speed. At last, in the payload cabin falling period, the east speed and north speed changed rapidly without rules.

**Figure 13:**
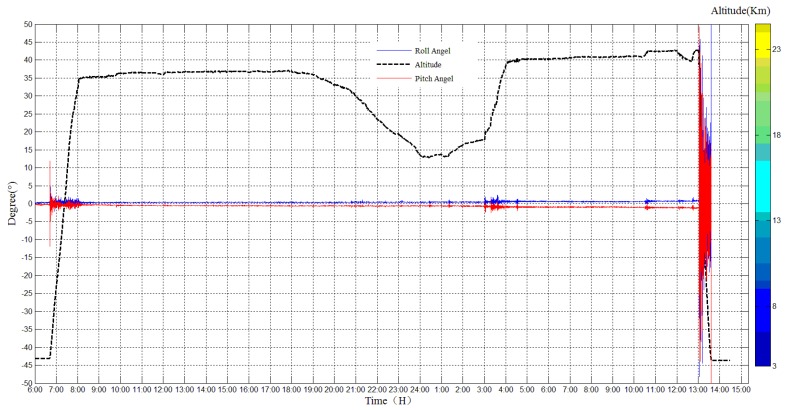
Roll angle and pitch angle in the flight experiment.

The angles of the payload cabin were monitored and reordered during the whole experiment, as shown in Figure 13, the blue line indicates roll angle and the red line indicates pitch angle. In the raising period, the roll angle and the pitch angle changed under the influence of the wind. However, in the zero-wind area, both the pitch angle and roll angle were very stable. In the falling period, the pitch angle and roll angle changed rapidly.

**Figure 14:**
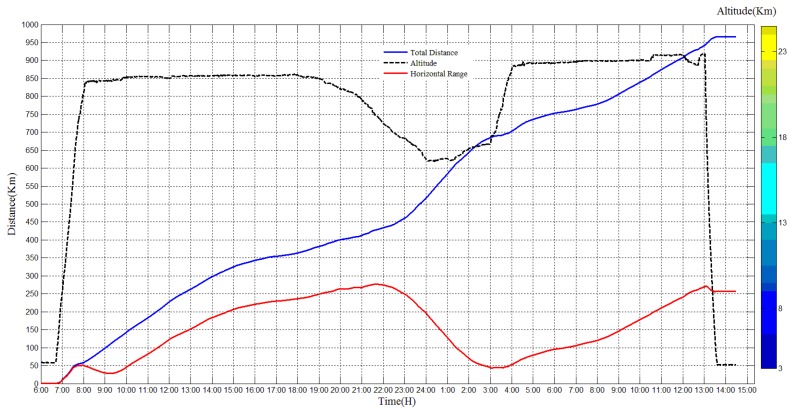
Total distance and horizontal range of the flight experiment.

The total distance and horizontal distance of the experiment are calculated using original position data, as shown in Figure 14, the blue line indicates the total distance, and the red line indicates the horizontal range. The total distance is more than 950 Km, and the horizontal range is about 170 Km. 

### 3.2. Temperature Sensor Result

**Figure 15:**
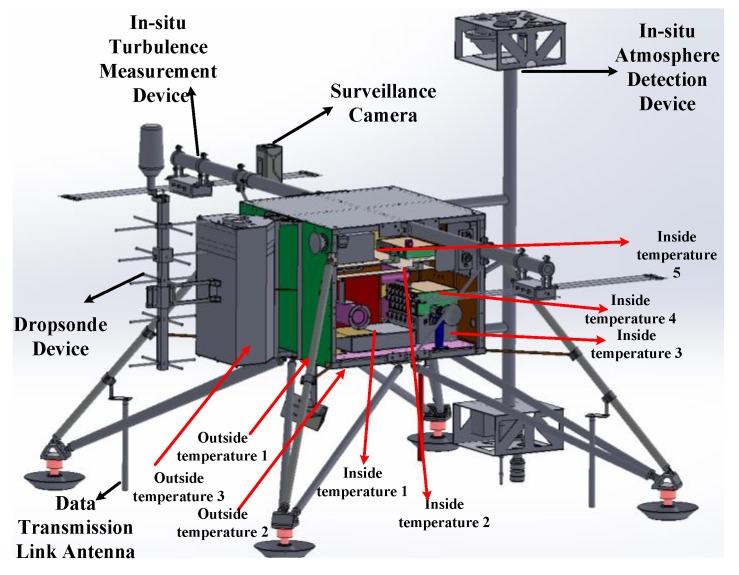
Temperature sensor location of the payload cabin.

The temperature sensors used in the experiment contain eight, and they were distributed inside and outside the payload cabin, as shown in Figure 15. Inside, temperature sensor 1 was placed near the data storage module, temperature sensor 2 was placed on the metal plane in the middle of the cabin, temperature sensor 3 was placed near the power supply module, temperature sensor 4 was placed near the main control module, and temperature sensor 5 was placed near the in-situ atmosphere detection device control module. Three outside temperature sensors were placed under the payload cabin, near the dropsonde device and on the payload cabin leg. 

**Figure 16:**
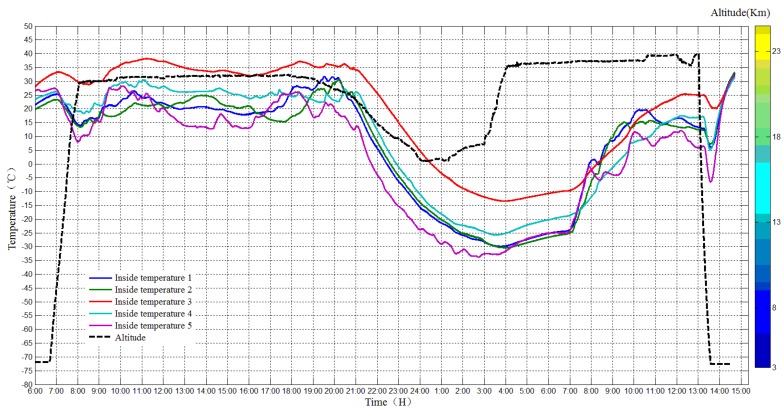
Temperature inside the payload cabin.

**Figure 17:**
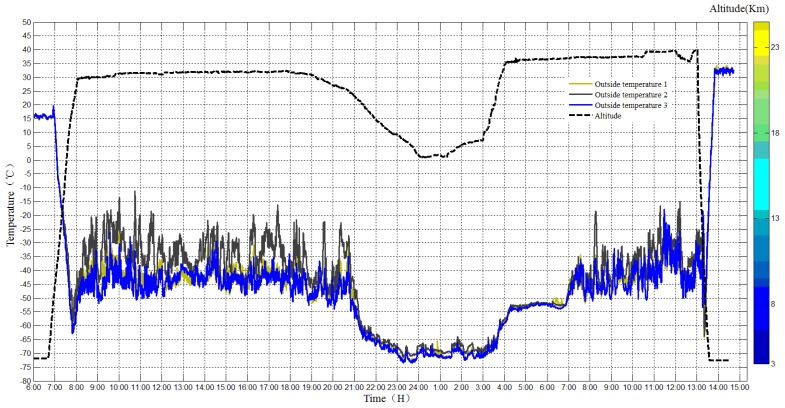
Temperature outside the payload cabin.

The temperature sensors inside and outside the payload cabin worked normally during the flight, and the temperature was monitored and reordered, as shown in Figure 16 and Figure 17. 

The temperature in the cabin during the daytime ranged from 10 to 30 °C, while the ambient temperature outside the payload cabin was −50 °C. However, the lowest temperature during the night in the cabin was −30 °C, while the ambient temperature outside the payload cabin was −70 °C. The temperatures of equipment in the payload cabin vary a lot since they have different energy consumption.

### 3.3. Image Surveillance Sensor Result

**Figure 18:**
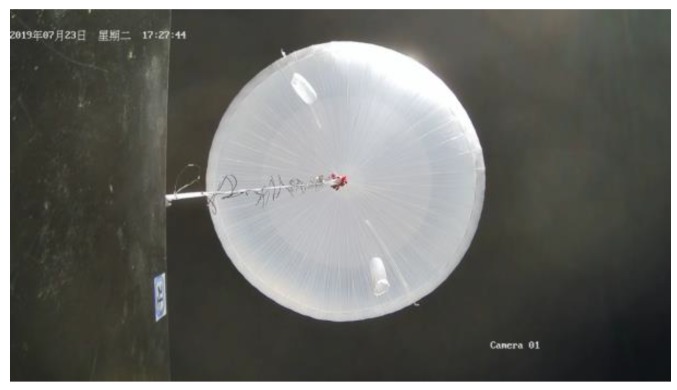
High-altitude balloon was fully inflated.

**Figure 19:**
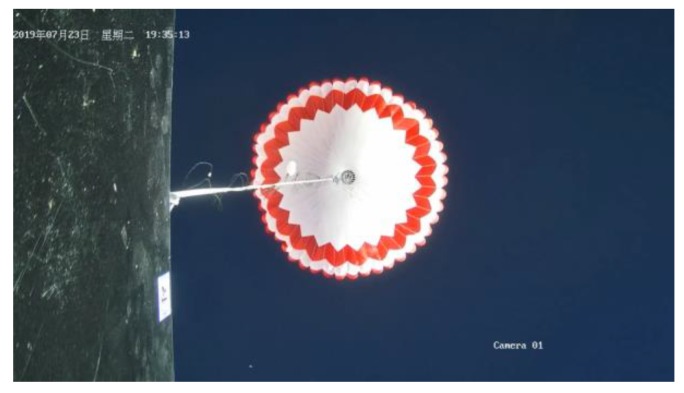
Parachute state during the falling period.

**Figure 20:**
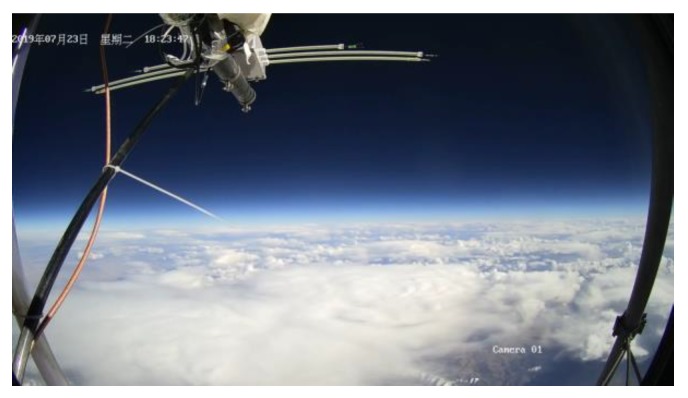
Image taken by the front view camera.

**Figure 21:**
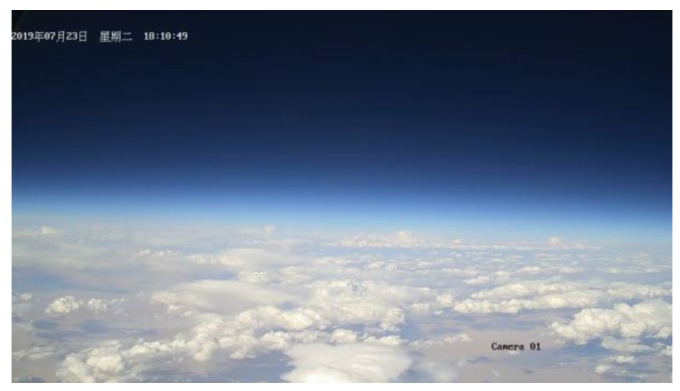
Image taken by the back-view camera.

The image surveillance cameras worked normally during the whole experiment and reordered many videos from different views. The upward surveillance camera monitored the whole process of the experiment, including raising process, flat flying period, cutting off process and falling process. Figure 18 shows the images taken by the upward surveillance camera in the afternoon. It can be seen that the high-altitude balloon was fully inflated and the balloon state was fine. Figure 19 shows the falling period after the balloon was cutting off and the payload cabin fell with gravity while the parachute was open. Figure 20 shows the picture taken by the surveillance camera looking forward, it can be seen that the in-situ detection payload was in the picture. Figure 21 shows the picture taken by the surveillance camera facing backward, and the image shows a beautiful view of the near space.

**Figure 22:**
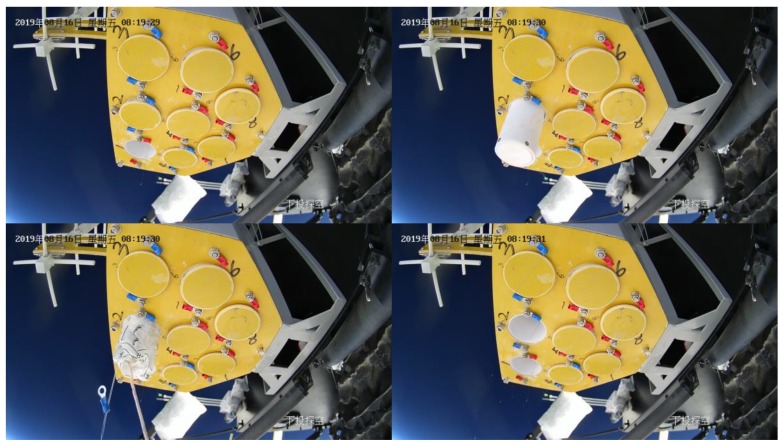
Images taken by the camera toward the payload.

**Figure 23:**
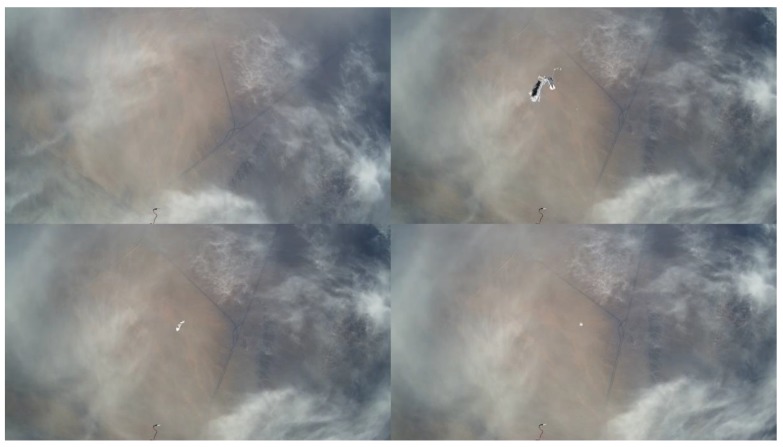
Images taken by the downward camera.

Figure 22 shows the images taken by camera towards the payload, and it recorded the breaking out process of the sounding tube. The dropsonde device can detect normal atmospheric parameters (temperature, humidity, wind direction and wind speed, air pressure) of vertical profiles during the falling period. As shown in Figure 22, the dropsonde device contains eight dropsonde modules, and the sensor data are sent to the control module through a 433 MHz transmission link. The control command is sent by satellite communication. Figure 23 shows the images taken by the downward camera, and it records the falling process of the sounding tube. There was a small parachute on each sounding tube to slow down the falling speed.

### 3.4. Work Current Sensor Result

**Figure 24:**
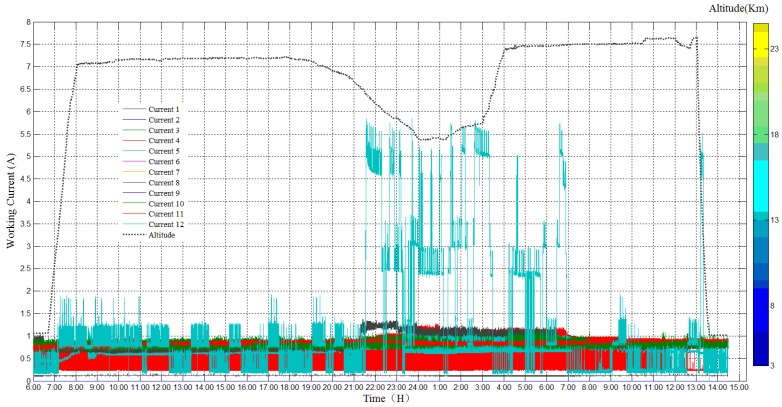
Current of all the equipment during the experiment.

The current sensor system worked normally during the whole experiment and reordered the current of all the equipment, as shown in Figure 24. The current of most of the equipment is around 1 A, only one payload changed significantly. Current 12 shows the current of dropsonde device; it changed largely since the dropsonde device was installed outside the payload cabin and it contained a warm-up module and consumes more energy. Other devices include a lightning imager, in-situ detection sensors, main control module, power supply module, surveillance camera module, data transmission module, GPS and IMU module, a data storage module, and so on.

## 4. Conclusions

The high-altitude balloon-based sensor system designed in this paper had participated in the near-space flight experiment, whose total flight time was 30 h and 53 min, and the horizontal flight time was 28 h and 58 min crossing the day and night. The high-altitude balloon-based sensor system had withstood the severe environmental of the near-space during day and night, providing accurate temperature measurement, real-time altitude position and attitude data acquisition, reliable current monitoring, and comprehensive video surveillance.

In the next three years, the high-altitude balloon-based sensor system developed in this paper will continue to participate in other experiments. The first experiment is a simulated meteorite landing experiment, which makes use of a high-altitude balloon platform, and discusses the possibility of life transmission between interstellar (interplanetary). The second experiment aims to understand problems such as climate change in parts of Asia by using a CO_2_ detection device and CH_4_ detection device. The third experiment uses a high-altitude balloon to carry the in-situ detection loads such as the atmospheric detector, electromagnetic detector, and neutron radiation detector to near space, carrying out the comprehensive observation. The fourth experiment uses a high-altitude balloon to carry biological exposure experimental device, UV spectrometer into near space, focusing on the UV radiation and its impact on representative organisms.

## Figures and Tables

**Table 1:** Inertial navigation sensor (INS) parts and the key specifications.

INS Part	Item	Specification
Position Accuracy	Horizontal(m) Vertical(m)	2 m 2.5 m
Velocity Accuracy	Horizontal/Vertical (m/s)	0.1 m/s
Attitude Accuracy	Roll/Pitch (°) Heading (°)	0.1° 0.5°
3-axis Accelerometers	Full Scale(g)	−16 g to 16 g
	Sampling rate (KHz)	4
	Gain stability (ppm)	1000
3-axis Gyroscopes	Full scale(°/s)	−450 °/s to 450 °/s
	Sampling rate (KHz)	10
	Gain stability (ppm)	500
3-axis Magnetometers	Full scale (Gauss)	−50 Gauss to 50 Gauss
	Sampling rate (Hz)	100
	Gain stability (%)	<0.5%
GNSS Receiver	Signal Tracking	GPS L1 C/A, GLONASS L10F QZSS L1 C/A, BeiDou B1
	Output Frequency (Hz)	5 Hz
Internal Barometric Altimeter	Resolution (Pa)	1.2 Pa
	Update rate (Hz)	100 Hz
